# Reported transfusion‐related acute lung injury associated with solvent/detergent plasma – A case series

**DOI:** 10.1111/trf.16822

**Published:** 2022-02-17

**Authors:** Robert B. Klanderman, Esther B. Bulle, Josephine W. M. Heijnen, Judith Allen, Ilse M. Purmer, Jean‐Louis H. Kerkhoffs, Johanna C. Wiersum‐Osselton, Alexander P. J. Vlaar

**Affiliations:** ^1^ Department of Intensive Care Amsterdam UMC, University of Amsterdam Amsterdam The Netherlands; ^2^ Laboratory of Experimental Intensive Care and Anesthesiology Amsterdam UMC, University of Amsterdam Amsterdam The Netherlands; ^3^ Department of Anesthesiology Amsterdam UMC, University of Amsterdam Amsterdam The Netherlands; ^4^ TRIP Hemovigilance and Biovigilance Office Leiden The Netherlands; ^5^ Department of Quality and Security HagaZiekenhuis The Hague The Netherlands; ^6^ Department of Intensive Care HagaZiekenhuis The Hague The Netherlands

**Keywords:** plasma, SDP, solvent/detergent, TRALI

## Abstract

**Background:**

Antibody‐mediated transfusion‐related acute lung injury (TRALI) is caused by donor HLA or HNA antibodies in plasma‐containing products. In the Netherlands 55,000 units of solvent/detergent plasma (SDP), a pooled plasma product, are transfused yearly. It's produced by combining plasma from hundreds of donors, diluting harmful antibodies. Due to a lack of reported cases following implementation, some have labeled SDP as “TRALI safe”.

**Study design and methods:**

Pulmonary transfusion reactions involving SDP reported to the Dutch national hemovigilance network in 2016–2019 were reviewed. Reporting hospitals were contacted for additional information, cases with TRALI and imputability definite, probable, or possible were included and informed consent was sought.

**Results:**

A total of three TRALI and nine TACO cases were reported involving SDP. The imputability of one TRALI case was revised from possible to unlikely and excluded; in one case no informed consent was obtained. We present a case description of TRALI following SDP transfusion in a 69‐year‐old male, 3 days following endovascular aortic aneurysm repair. The patient received one unit of SDP to correct a heparin‐induced coagulopathy, prior to removal of a spinal catheter post‐operatively. Within five hours he developed hypoxemic respiratory failure requiring intubation, hypotension, bilateral chest infiltrates, and leucopenia. The patient made a full recovery.

**Conclusion:**

This case of TRALI, following transfusion of a single unit of SDP to a patient without ARDS risk factors, demonstrates that TRALI can occur with this product. Clinicians should remain vigilant and continue to report suspected cases, to help further understanding of SDP‐associated TRALI.

## 1 INTRODUCTION

While transfusion can be lifesaving, it also has life‐threatening adverse effects including transfusion‐related acute lung injury (TRALI). TRALI is a syndrome of acute onset pulmonary permeability edema. It often leads to intensive care (ICU) admission and a need for mechanical ventilation and is associated with a 10%–15% fatality rate in hospitalized patients,^1^ and up to 50% in the critically ill.^2^ Antibody‐mediated TRALI is caused by anti‐HLA and HNA antibodies from donors, preserved and stored in plasma‐containing blood products. Introduction of male‐only plasma products, limiting alloantibodies prevalent in (multi)parous women, reduced the incidence of TRALI.^3^, ^4^ Further advances in plasma manufacturing, by solvent/detergent treatment of pooled plasma (SDP), allow these harmful antibodies to be diluted below the detection limit by combining 300 to 500 or more donations.^5^ Approximately 55 thousand units of SDP are transfused yearly in the Netherlands and it has been adopted as a primary plasma product by numerous countries over past years. Since its introduction, the incidence of TRALI has plummeted.

TRALI develops according to a two‐hit event threshold model.^6^ The first hit is a patient's underlying condition, such as sepsis or trauma, which primes neutrophils in the lungs, lowering their activation threshold. Subsequent transfusion of anti‐HLA and HNA antibodies, specifically in plasma‐containing blood products, can activate these neutrophils leading to fluid extravasation which causes respiratory distress. The threshold to activate primed neutrophils is lower in patients with a more severe first hit; that is, neutrophil activation following a second hit will require fewer antibodies.^7^

SDP has been described as “devoid of antibodies”,^8^ and SDP has “abolished” antibody‐mediated TRALI.^9^ However, dilution of the harmful antibodies does not remove antibodies, and TRALI remains a clinical diagnosis that does not require HLA and/or HNA antibodies. We investigated pulmonary transfusion reactions (cases classified as either TRALI or transfusion‐associated circulatory overload [TACO]) involving SDP reported to the Dutch national hemovigilance office and present a case of TRALI after a single unit of SDP.

## 2 MATERIALS AND METHODS

This study was approved by the medical ethics committee (Amsterdam University Medical Center, location AMC, reference number: W18_432 # 19.016). All reported cases of TRALI and transfusion‐associated circulatory overload (TACO) involving SDP (Omniplasma®, Octapharma GmbH – *Germany*) submitted to TRIP (transfusion and transplantation reactions in patients), the Dutch hemovigilance office between January 1st, 2016 and December 31st, 2019 were reviewed. TRIP's working methods have been reported elsewhere.^10^

Quarantined, male‐only fresh frozen plasma was the national standard plasma product for transfusion prior to the implementation of SDP. From 2014–2016 the national blood establishment, Sanquin progressively rolled out SDP (Omniplasma®). The production process is identical to that of Octaplas® LG (Octapharma GmbH – *Germany*), however, donors are exclusively Dutch unpaid male volunteers. A major advantage of SDP products is the dilution of anti‐HLA and anti‐HNA antibodies. Moreover, donor plasma is known to contain soluble HLA antigen which theoretically captures and neutralizes harmful antibodies.^11^

TRIP identified eligible cases during the included study period and contacted the hospitals through the hemovigilance officers with a request for additional information about the reaction, using a questionnaire pre‐filled with details from the original report to TRIP. Due to privacy laws and doctor‐patient confidentiality, cases from TRIP were analyzed anonymously by R.K., E.B. and A.V. We sought to obtain the informed consent of TRALI patients through their treating physicians, to perform a full chart review as well as permission to publish information.

## 3 RESULTS

A total of 12 pulmonary adverse transfusion reactions (nine TACO, three TRALI) were reported to TRIP in association with SDP during the inclusion period; there were no cases of transfusion‐associated dyspnea (TAD). In nine cases (75%) hospitals supplied additional information. Reevaluation of the hemovigilance reports in combination with additional data supplied did not result in a change of classification of TACO to TRALI or vice versa. In total, we have a case series of three TRALI patients involving SDP plasma. In one case of TRALI, the imputability was changed from possible to unlikely and excluded from more detailed presentation in the current case series. We did not receive informed consent to present patient details for one case. We, therefore, present one case of TRALI after SDP plasma transfusion in more detail below.

### 3.1 Case presentation

A 69‐year‐old male was admitted post‐operatively to the ICU, as a planned admission, after an elective endovascular aortic repair (EVAR). Relevant past medical history included an inguinal hernia repair and 55 pack‐years of smoking. Under general anesthesia, a fenestrated EVAR of a thoraco‐abdominal aneurysm involving the coeliac trunk, the superior mesenteric artery, and both renal arteries, was performed during a five‐and‐a‐half‐hour procedure. An intrathecal catheter was inserted pre‐operatively to allow for drainage of cerebrospinal fluid intra‐ and post‐operatively to alleviate pressure on the spinal cord and improve perfusion pressure. An inherent risk of EVAR procedures is the possibility of spinal cord ischemia leading to paralysis after stenting of the artery of Adamkiewicz. There were no complications intra‐operatively, with minimal blood loss (200 mL); intraoperative mechanical ventilation was unremarkable. The patient was admitted to the ICU for post‐operative care. The patient arrived extubated, had documented normal vesicular breathing sounds, did not require vasopressors, and had a maximum score on the Glasgow Come Scale.

A continuous heparin infusion, readily adjustable and short‐acting (target activated partial thromboplastin time [aPTT]: 60–90 s), was started to prevent in‐stent thrombus formation as a bridge to oral anticoagulation while the spinal catheter was in‐situ. Notably, insertion or removal of a spinal catheter, especially under anticoagulant medication, carries a risk of a spinal hematoma developing.^12^ The hematoma can press on the spinal cord and result in paralysis and constitutes a neurosurgical emergency.

The first and second post‐operative days were unremarkable, with the patient having no signs or symptoms of the spinal cord, hepatic, or bowel ischemia, nor acute kidney injury. On the third post‐operative day, in order to remove the spinal catheter, the heparin infusion was stopped at 4 AM. Some bleeding had been noticed around the spinal catheter insertion site prior to removal of the catheter as well as from the inguinal surgical site in combination with an aPTT of 48 s (reference: 30–40 s), a single unit of SDP was transfused to correct the coagulopathy. There is no guidance on specific coagulation factor levels or aPTT values prior to the removal of a catheter.^12^

**FIGURE 1:**
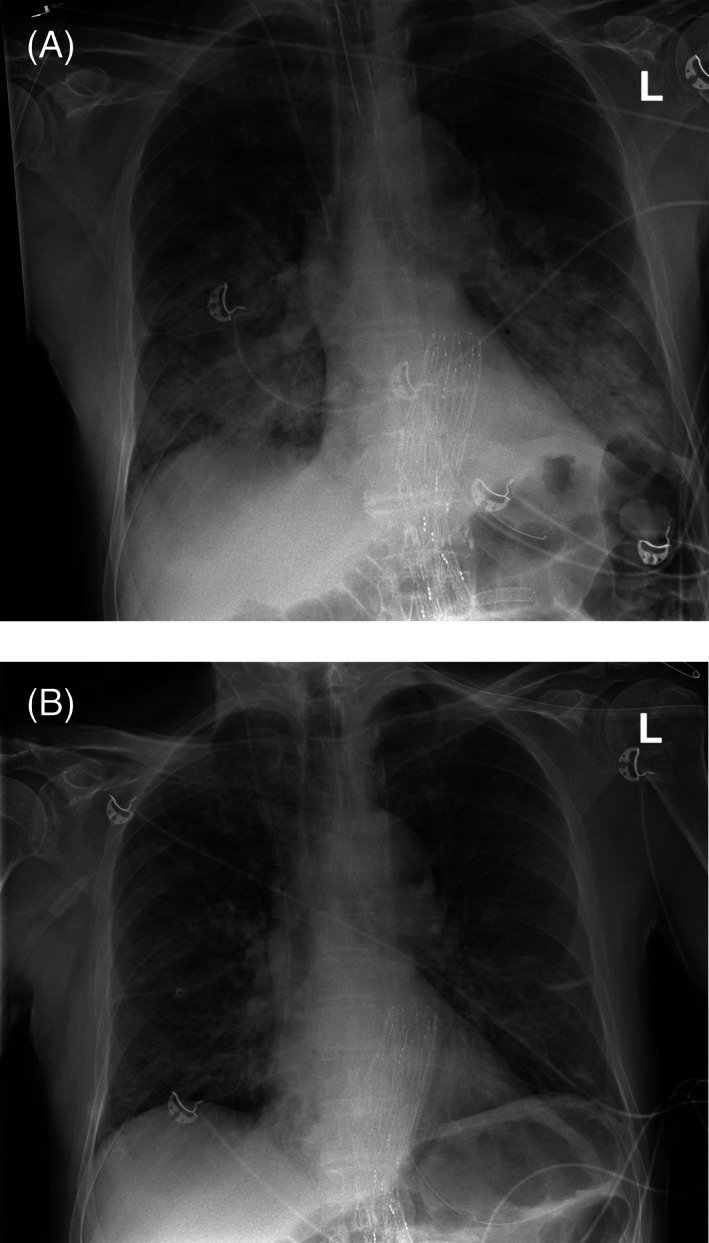
Case 1 chest x‐ray. *Panel A*: 3 h:15 min after start of transfusion. Perihilar alveolar edema is present, with bilateral patchy consolidations visible. *Panel B*: 2‐days post‐transfusion: Improvement of consolidations in the lung fields bilaterally

The transfusion was started at 10:45 on post‐operative day three. At 11:30 physical examination documented a respiratory rate of 19 breaths/minute and normal breathing sounds. At 13:30 the patient, while not complaining of dyspnea had a measured SpO_2_ of 80% with desaturations to 75%. He was able to speak sentences, though respiratory frequency had increased to 32 breaths/min, with lung examination revealing decreased breath sounds over the lower lung fields bilaterally. He became tachycardiac (150 beats/min) and blood pressure decreased to 80/47 mmHg. The differential diagnosis included TRALI and aspiration pneumonia (Table 1), however, no evidence was found for the latter. A chest x‐ray revealed bilateral patchy consolidations with alveolar edema (Figure 1). Antibiotics and non‐invasive positive pressure ventilation (NIV) were started, however, the patient developed hypoxic respiratory failure and rapid sequence intubation was performed. The patient initially required a 70% FiO_2_ to achieve a 9.7 kPa PaO_2_ (PaO_2_/FiO_2_‐ratio: 137), however, FiO_2_ could be rapidly lowered to 35% over 6 h. Profound leucopenia (1.4•10^9^/L) was seen in laboratory analysis 3 h after transfusion.

**TABLE 1:** Case presentation

Characteristics	Pre‐transfusion 10:00 AM	Post‐transfusion 2:00 PM
Initial symptom	Increased respiratory rate and hypoxemia	
Time to onset of symptoms	2 h	
Respiratory rate (breaths/min)	12	32
Oxygen saturation	98%	75%
PaO_2_ (kPa)	—	7.1
Supplemental O_2_	No	NIV – FiO_2_ 80%
Mechanical ventilation required	No	Yes
PaO_2_/FiO_2_‐ratio	—	67
Heart rate (beats/min):	78	150
Blood pressure (mmHg)	97/88	80/47
Vasopressors	No	Yes
Temperature (°C)	37.2	37.0
Lung auscultation	Clear breath sounds	Decreased basal breath sounds bilaterally
Leucocytes (×10^9^)	16.2	1.4
Chest X‐ray	—	Perihilar alveolar edema & spotty consolidations
Severity		Grade 3^a^
ICU length of stay		4 days
Duration of mechanical ventilation		24 h

Abbreviations: NIV, Non‐invasive ventilation; FiO_2_, Fraction of inspired oxygen; ICU, Intensive care unit.

^a^ Severity grade according to TRIP and ISBT‐IHN definitions, see https://www.isbtweb.org/fileadmin/user_upload/Proposed_definitions_2011_surveillance_non_infectious_adverse_reactions_haemovigilance_incl_TRALI_correction_2013_TACO_correction_2018.pdf.

The patient was extubated after 24 h of mechanical ventilation and discharged from the ICU with six liters of oxygen via nasal cannula on post‐operative day four. He was discharged home on post‐operative day 11. The case was reported to the hospital's hemovigilance officer. No anti‐HLA and anti‐HNA antibody titers were performed on either patient blood samples or the SDP unit.

## 4 DISCUSSION

This is the first case series of TRALI developing after transfusion of SDP plasma. The case presented has a likely imputability and is associated with a transfusion of a single unit of SDP. The case conforms to the 2019 consensus redefinition of TRALI type I,^13^ where there is a clear relationship between transfusion of SDP and within 6 h: (a) an acute onset; (b) hypoxemia with a PaO_2_/FiO_2_‐ratio < 300 as well as an SpO_2_ < 90% on room air; (c) no temporal relationship to an alternative risk factor for ARDS considering the transfusion reaction occurred on post‐operative day three.

To date, only three cases of TRALI have been associated with SDP. The first case was described in the 2016 annual TRIP report^14^; no details of the case are provided and following hemovigilance expert review it was judged that that TACO could not be ruled out. The second case was included in the 2019 annual TRIP report^15^ and is described in detail here. A third case is described in the annual 2018 TRIP report, but we excluded this case based on adjusted imputability due to the complex nature of the case which included pulmonary edema following massive transfusion and multiple episodes of cardiac arrest. The lack of published cases so far shows that passive reporting of TRALI due to plasma transfusion is an extremely rare occurrence, with over 55 thousand units of SDP transfused every year in the Netherlands alone. The described case shows TRALI can occur, and medical personnel involved in blood transfusions need to be alert and aware of the potentially severe complications.

The pathophysiology of TRALI following plasma transfusion is regarded as antibody‐mediated,^16^ as opposed to cellular transfusion products which can result in both antibody‐mediated, and non‐antibody‐mediated TRALI due to soluble mediators.^17^ Even though antibody titers are undetectably low in SDP,^5^ dilution, by definition, never removes antibodies. Antibodies in SDP can therefore not be ruled out as the second hit. Unfortunately, no antibody titers were performed in this patient nor in the SDP unit. TRALI, as seen in the case described, however, is a clinical diagnosis and antibodies are not required to make the diagnosis.

It is surprising there are not more reported cases of TRALI, as some patients will develop acute respiratory distress syndrome within 6 h following transfusion, even if unrelated to transfusion. The lack of detectable antibodies in SDP may have assuaged fear of TRALI as a complication of plasma transfusion and reduced vigilance, or it may even function as a self‐fulfilling prophecy wherein a diagnosis of TRALI is refuted due to the absence of measurable antibodies.

Immunological workup and donor testing in SDP‐associated TRALI is not feasible. Fresh frozen plasma allowed for straightforward donor identification, and testing could guide donor deferral in order to prevent TRALI.^18^ Identification of one or more culprit donors amongst 300 to over 500 pooled plasma donations is unfeasible. To date, the current assays cannot detect harmful antibodies in SDP after pooling. Improved testing techniques and methods for antibody inactivation warrant further investigation if we want to rule out the possibility of antibody‐mediated TRALI associated with this product. Alternative pathophysiological mechanisms include “reverse” TRALI.^19^ In this case, not donor antibodies, but antigens, are transfused into a sensitized patient with multiple antibodies, thereby activating neutrophils. It is unknown whether other soluble mediators in SDP or reactions with storage solution can contribute to the development of TRALI, and further recognition and reporting of cases will aid research efforts. The current report sets the stage to investigate the safety of SDP in patients at risk for TRALI in a prospective manner.

## 5 CONCLUSIONS

The reported number of TRALI cases associated with SDP is very low. Here one case described in detail demonstrates that TRALI can occur following a single‐unit transfusion of SDP. Clinicians should remain alert to the risk of TRALI with SDP as with other blood components and continue to report cases of respiratory distress after transfusion of SDP.

## CONFLICT OF INTEREST

The authors declare that they have no competing interests.
